# So, what about *P*?

**DOI:** 10.3325/cmj.2019.60.469

**Published:** 2019-10

**Authors:** Vladimir Trkulja, Pero Hrabač

**Affiliations:** 1 Department of Pharmacology, Zagreb University School of Medicine, Zagreb, Croatia *vtrkulja@mef.hr*; 2 Department of Medical Statistics, Epidemiology, and Medical Informatics, “Andrija Štampar” School of Public Health, University of Zagreb School of Medicine, Zagreb, Croatia

The 2016 statement by the American Statistical Association (ASA) (1) and a subsequent ASA editorial in March 2019 (2), both accompanied by extensive supplements illustrating a wide-ranging discussion among the leading contemporary statisticians, address numerous facets of scientific reasoning based on the concept of “*p*-values and statistical significance.” Both of these articles attempted to straighten-out distortions and misinterpretations of the original concept that have occurred over the decades, but also to reconsider *P* values as one of the core elements of the modern statistical science. They have attracted much attention of the scientific community (not only statisticians) (2), although for people without a solid statistical education it may be difficult to grasp their full meaning. On the other hand, one does not necessarily need to comprehensively perceive the conceptual and theoretical complexity in order to better understand and interpret one’s own research or other people’s data. Both editorials (1,2), and at least some of the accompanying articles (eg, 3,4 – a subjective choice!), provide an informative overview of the historical context and point-by-point lists of misinterpretations of *P* values, ie, of reasoning that should be abandoned, as well as of reasoning that should be adopted (and why), in a way that should be understandable to any (statistically) lay person, for example to “average medical doctors” like us.

We guess that, overwhelmed by daily workload, most of our colleagues are not very likely to have time (and interest) to get a deeper insight into the on-going developments regarding “statistical tests and *p*-values,” the concepts that, in most cases, have been “engraved” into our perception of statistics. We would therefore like to try to outline, to the best our understanding, the way of thinking that has been advocated (1,2). For this purpose, we use a rather simple hypothetical scenario that exemplifies a test of a null hypothesis [one of the statistical hypotheses, ie, the one with which “it all started” in the early 20th century (1-4)].

## A hypothetical example

A new treatment (T) for generalized anxiety disorder (GAD) is being developed based on a mechanistic understanding of its effects on monoaminergic and cannabinoid neurotransmitter systems and their involvement in anxiety. Its non-clinical safety pharmacology and toxicology profile is satisfactory and it shows beneficial effects in animal paradigms. In the early-phase clinical development, the aim is to evaluate whether T indeed has the potential to alleviate symptoms in adults with GAD. This would mean reduction of symptom severity as quantified by some validated instrument [eg, Hamilton Anxiety rating scale (HAM-A), a standardly recommended instrument (5)], in patients treated with T over a certain period of time. This reduction should be attributed to the treatment with T rather than to spontaneous oscillations in disease severity (natural course of the disease) and/or to an “expectation” arising from the sole idea of receiving a treatment. Therefore, this initial evaluation needs to be in the form of a clinical experiment – randomized controlled trial, double-blind, where the control needs to be a matching placebo (PBO), which should convey the same level of “expectation” as T. The trial needs to be long enough to allow for the onset of the (presumed) effect but not too long, as this could be ethically doubtful (not treating patients who suffer); GAD has to be clearly clinically obvious (in order to observe the presumed effect) but not too severe as this also could be ethically doubtful. Hence, an 8-week trial is conceived that should include patients with moderate-to-moderately severe GAD, eg, HAM-A scores 20-32 (6). Based on what is known about anxiety, the roles of monoaminergic and cannabinoid systems, mechanistic properties of T, and limited predictivity of animal paradigms for human anxiety, plausible outcomes of such a trial could be 3-fold: T could reduce anxiety, it could worsen anxiety, and it could be “inert”, ie, neither reduce it nor worsen it. In a more formal language, this trial represents a set-up for a test of a null hypothesis [commonly abbreviated as NHST – null hypothesis significance test (1,2)]. The word “null” means “no effect” or “there is no benefit and no harm (no effect) of T regarding the severity of GAD,” or “the true (population) effect of T on severity of GAD=0.” But, this setting has a rather extensive meaning. The hypothesis (the null) is defined *a priori*, ie, before the trial, as a part of a model that includes a number of other hypotheses that are *a priori* considered to be true, for example: that the included participants are indeed a random sample from the population; that they are assigned (ie, properly randomized) to T or PBO without prejudice; that double-blinding in the trial is kept throughout, so that no biases in provided care and other measures (and expectations) are likely to occur; that HAM-A indeed adequately quantifies anxiety in GAD patients; that there is no interference (pharmacodynamic or pharmacokinetic) with other treatments; that compliance with the assigned treatments is adequate; that all relevant data are adequately captured; that data analysis procedure fits the numerical properties of the data, and so on (and many times “and so on…”). A rather complex model, but, based on the data that are to be collected, only one assumption of the model will be tested: the hypothesis about the effect of T, ie, the null hypothesis. To rephrase it – once collected, data will be contrasted to the entire model, yet a conclusion of the test will pertain to only one of its hypotheses (the null). The basic outline of this hypothetical trial is given in Box 1.

Box 1 **The hypothetical trial – “technical” aspects and theoretical background**

**Figure 1:**
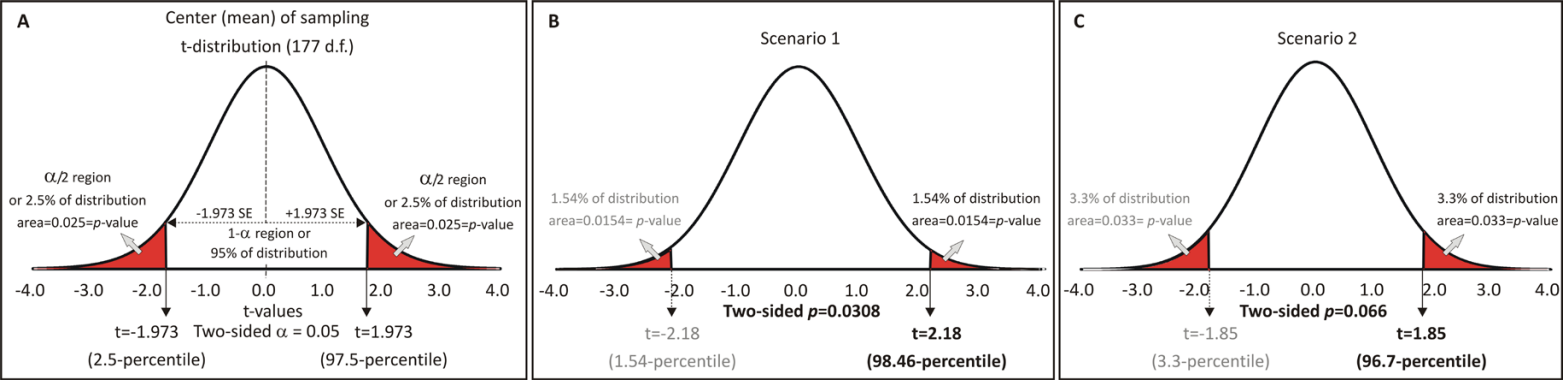
Student’s *t*-distribution (probability density function) with 177 degrees of freedom pertinent to the hypothetical trial. (**A**) Outline and important elements. (**B**) Position of the *t*-score and *P*-value in the scenario 1. (**C)** Position of the *t*-score and *P* value in the scenario 2.

In clinical trials, adults with GAD experience considerable average reductions in symptom severity with any treatment, including placebo, at least over the initial period (7,8). Functional pharmacological anxiolytics provide somewhat greater (average) reductions than placebo, by around 2-4 HAM-A scale points (8,9). The trial aimed to obtain a reasonably precise estimate about the effect of T, ie, a confidence interval (CI) around the effect measure that would not be too wide. The primary effect measure in this trial is difference T-PBO in the mean HAM-A score reduction at week 8 vs the baseline value, adjusted for the baseline HAM-A score (since any “change vs baseline” is largely affected by the baseline value), ie, adjusted mean difference T-PBO. It was decided that it would be satisfactory to obtain an estimate in which the distance between the lower and upper limits of the 95% CI around it would not be greater than 6 score points (for example, for a T-PBO difference of 0, the CI would extend from -3 to +3). A review of similar trials indicated that the standard deviations (SD) of score reductions in patients treated with active treatments and placebo are closely similar and mostly (at 6-10 weeks) range between 7 and 12 score points (8,9). Therefore, it was reasonable to assume that T- and PBO-treated patients would have equal SDs of 10 score points. Based on these assumptions, it was calculated that the desired width of the 95% CI around the T-PBO difference could be achieved if 180 patients were enrolled and randomized 1:1 to T and PBO (and provided data). We outline the theoretical background for the estimation of the mean difference T-PBO and for the formal test of the null hypothesis [the concept of sampling distribution was outlined in the previous issue (10)] in this hypothetical trial. The mean change in HAM-A score with T and mean change with PBO are independent normal random variables (their population distribution is normal), hence their difference is a normal variable. Therefore, the sampling distribution needed for the calculations would be a normal distribution. However, to strictly follow the statistical theory, a more appropriate sampling distribution is the *t*-distribution. Namely, the mean difference T-PBO (the effect, 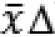)that is to be determined in the sample is an estimate of the mean (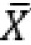) of the sampling distribution of 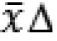, and since 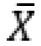 = m (mean of the population distribution), in this way an estimate of the true (population) T-PBO difference is obtained. What is still needed in order to proceed is the information on the measure of the spread of the sampling distribution. As the spread is typically unknown, it has to be estimated from the sample in the form of a standard error (SE) of the effect. It could be shown that SE from the sample overestimates the SD of the normal distribution when the sample is small and becomes closer to the population value as the sample increases. Student’s *t*-distribution (named after WS Gossett’s pen name “Student”) takes into account this uncertainty about the measure of the spread. Hence, the *t*-distribution is a family of distributions: like the normal distribution, they are bell-shaped probability density functions, symmetrical around 0 but differ from the normal distribution in the sense that, with smaller samples, they are “wider,” flatter around the mean, with “thicker” tails. Already with samples of around 30, the *t*-distribution is highly similar to the normal distribution, but it would be exactly identical only with infinitely large samples. Discrepancies between the *t* and normal distribution are important with small samples, but in theory, a sampling *t*-distribution of a mean difference would be used always when its spread needs to be estimated from the sample. Also, to be exact, the shape of the *t*-distribution is actually determined by the degrees of freedom (d.f.). D.f. in a statistical model refer to the number of observations that are free to vary in the process of estimation of the population value (11,12). In the current hypothetical trial with 180 participants (that would actually be enrolled and would complete the trial), the sampling *t*-distribution of the estimated effect would have 177 d.f. – one d.f. is “used” by the fact that we first need to determine mean HAM-A change with T; another one is “used” for the mean HAM-A change with PBO; and the third d.f. is “used” for the baseline HAM-A score (a covariate). Hence, 3 d.f. are “used” by the elements needed in the calculation of the effect, and the respective sampling distribution has 177 d.f. – such a distribution is illustrated in Figure 1A. It is important to have in mind what it represents: if a large number of repeated samples (patients) of the same size (180, randomized 1:1) from the same population (GAD patients with HAM-A score 20-32) would be taken, and T-PBO mean difference estimated in the same way, by chance (sampling variation), not only that each mean difference (an estimate of 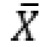) but also each SE (estimate of standard deviation) would somewhat differ from the others. Hence, estimates of the mean difference (from each such hypothetical repeated trial) need to be standardized as:

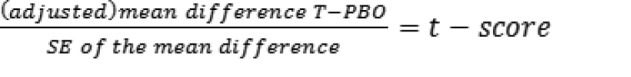

By this step, all these (hypothetical) estimates can be considered jointly, and their distribution is actually a distribution of *t*-scores. For any given variability in the sample (and SE of the mean difference), a larger effect would yield a larger *t*-score, and a smaller effect would yield a smaller *t*-score; for any given effect, a smaller SE would result in a higher *t*-score and a larger SE would result in a smaller *t*-score.

Figure 1A demonstrates: a) this is a bell-shaped probability density function (pdf) symmetrical around its mean (0); b) area under the pdf = 1.0, ie, it “covers” 100% of the probability; c)one detail indicates that this *t*-distribution with 177 d.f. slightly “deflects” from the normal distribution: the middle 95% of the distribution, ie, the area from its 2.5th to 97.5th percentile is defined as mean ±1.973 standard deviations (ie, its estimate, SE), while in the case of the normal distribution, the middle 95% extend within ±1.96 standard deviations from the mean; d) the 2.5% of *t*-scores that are above the 97.5th percentile (in this specific *t*-distribution>*t*-score of 1.973) and 2.5% of *t-*scores that are below the 2.5th percentile (in this specific *t*-distribution<*t*-scores of -1.973) are considered to be the extreme values in the distribution; e) this view that a total of 5% of the values in the distribution are considered extremes has existed from the very beginning of the concept of statistical tests and was defined largely arbitrarily but not without a reason (see 3 for the historical perspective); f) the criterion about a cut-off value pertaining to which part of the values are extremes is called α level. Since in symmetrical distributions a half of the extremes are above the 97.5th percentile and a half of the extremes are below the 2.5th percentile, the overall α level is designated as two-sided α level. The cumulative region of extreme values is called α region [(α/2) + (α/2)], while the middle region of the distribution is called 1-α region; g) the value of the area under the curve above the 97.5th percentile of the *t*-score distribution is 0.025 (or 2.5%) and represents the cumulative probability of such extreme values (or the *P* value), and the area under the curve below the 2.5th percentile of the *t*-score distribution (0.025 or 2.5%) is the cumulative probability of such extreme values (*P* value) in the “other direction.” Therefore, with the *a priori* defined “cut-off”, ie, α 0.05 – 5% of the values in the distribution are considered extremes. This general rule applies to any *t*-distribution [any number of d.f. – but with a different number of d.f., critical percentile (2.5th and 97.5th) *t*-score values will differ], normal distribution (extremes are z-scores lower than -1.96 and higher than 1.96), chi^2^-square, or any other sampling distribution.

In the null hypothesis test in this hypothetical trial, calculated adjusted mean difference T-PBO and its SE would be converted into a *t*-score, which would be “contrasted” to the respective *t*-distribution with 177 d.f. (Figure 1A) and its corresponding percentile value would be determined. If the *t*-score has a positive sign, the area under the pdf from its percentile value to the right is determined, while if it has a negative sign, the area from its percentile value to the left is determined. In either case, this area is multiplied by 2, and the resulting value is a two-sided *P* value. The two-sided *P* value is used because the null hypothesis in essence is a “two-sided hypothesis:” it reads “T-PBO=0”, but it makes no assumptions about the direction of a possible T-PBO difference (>0 or <0) (11,12). As indicated in the main text, the *a priori* hypothesis in this trial was intentionally defined as a null hypothesis: biologically, it is plausible that a difference (the effect) could go to either direction. Hence, numerical data from the entire trial would be “summarized” in a *P* value, which is used to evaluate to what extent the observed data (the sample mean difference) are compatible with the *a priori* null hypothesis (population difference = 0) (1,2,4). *P* value may extend between 0 and 1. If one would assume that all the assumptions, beyond the null hypothesis, of the *a priori* model that the trial subsumes are indeed correct, then, specifically in respect to the null hypothesis, *P* values in a way “quantify” the extent of compatibility of the observed effect and the null (effect = 0): *P* = 1 would mean full compatibility, *P* =  would mean no compatibility, and the values between them would mean various levels of (in)compatibility (1,2,4). Under the same circumstances, if the null hypothesis is true, any deflection of the observed effect from 0 would be due to a chance alone; under these circumstances, in terms of probability, a particular *P* value says: this is the probability of the observed effect, or a more extreme one, if the null hypothesis is true (4). For example, *P* value = 0.200 means “under the condition that all of the hypotheses inherent to the model are true, including the null hypothesis, the probability of the observed effect, or a more extreme one, is 20%.” This could be also rephrased as “under the condition that all of the hypotheses inherent to the model are true, including the null hypothesis, if exactly the same entire procedure (number of participants, trial design, etc) is repeated a large number of times in independent random samples from the same population, an effect of this size, or a more extreme one, will be obtained in 20% of the cases.” *P* values may be viewed also as a “surprise factor” (4,13,14), indicating how surprising or unexpected the observed effect is, if the null is true (under the condition that all other assumptions of the model are correct). For example, *P* = 0.010 would indicate a rather poor compatibility of the observed effect with the null, under the above circumstances, and would thus indicate that the observed effect is in a way surprising. Analogously, *P* = 0.500 would indicate a considerable compatibility of the observed effect with the null, and if the null is true (under the condition of correct other assumptions), the observed or a more extreme effect will be observed 50% of the time, ie, it will not be particularly surprising. It should be noted that all these considerations about *P* value and its “relationship” with the null hypothesis are made under the assumption that all other hypotheses in the model are correct. However, the fact is that a “low” or a “high” *P* value might be low or high not because of a real (in)compatibility of the observed effect with the null hypothesis, but because any one or more of the assumptions that constitute the *a priori* model – do not hold true. Therefore, an interpretation of a *P* value has to account for the fact that the observed data are contrasted against the entire *a priori* model, not only against the null hypothesis (1,2,4).

ASA statement and the subsequent editorial (1,2) particularly emphasized the distortions in the interpretation of the concept of the frequentist hypothesis tests and *P* values that have occurred over time. The idea of α level (*a priori* 5%) introduced at the early stages of the development of the concept (3), which was meant to indicate that, under ideal conditions elaborated above, *P* values ≤0.05 would indicate an effect with a particularly doubtful compatibility with the null hypothesis, has been distorted into an unjustified dichotomization, where *P* ≤ 0.05 was turned into a universal proof of an effect and *P* > 0.05 into a proof of the lack of it (a practice neither initially conceived nor scientifically plausible), with all the further misconceptions and poor practices, from data reporting, interpretation (eg, if *P* ≤ 0.05, effect is practically relevant), disregard of the other aspects of the *a priori* model, publication polices, and many others (see 1, 2, 4 for point-by-point elements of malpractice). Although a number of criticisms have addressed the concept itself, many of them are not justified (15). What primarily appears to have been flawed is the way of its application. The emphasis has been put on appropriate interpretation of the results of statistical tests and associated *P* values that should always be considered within the context in which they were generated (eg, characteristics of the study, pre-existing knowledge, specificities of the scientific discipline, actual method of data analysis) (1,2,4,14-17).

Table 1 summarizes two hypothetical outcomes (scenario 1, scenario 2). In both cases, the targeted number of participants was enrolled (with only a slight imbalance between treatment arms), treated, and completed the trial, the point-estimate of the mean T-PBO difference was in agreement with published data on functional anxiolytics (indicating greater symptom reduction with T than with PBO), and the attained width of the 95% CIs around the estimate was in line with the expectations (see Box 1). In the scenario 1, *t*-score is 2.18 and is at the 98.5th percentile of the respective *t*-distribution (Figure 1B), resulting in *P* = 0.0308 (Table 1). In agreement with this, the entire 95% CI around the estimate is above 0.0. Under the condition that all the assumptions of the model (ie, those beyond the null hypothesis) are true, this effect is hardly compatible with the null hypothesis. Namely, if (in addition) the null were true, the effect of this size or a larger one would be observed only 3.1% of the time. The lower limit of the 95% CI is not “very far” from 0.0 (Table 1), but the interval is not too precise (in the sense of its width), which is in line with the expectations and the planned sample size. There appears to be no unexpected variability in the data. Central randomization was performed, double-blinding was broken only after data analysis was completed, there was no attrition, and the compliance was high (>90% dispensed/consumed). Patient comorbidity was minimal, typical for GAD patients, and was well balanced between the treatment arms. The results on two additional rating scales used as secondary measures (General Anxiety Disorder-7 and Clinical Global Impression- Improvement) were in the same direction as that on the primary instrument (HAM-A). All this supports the view that the observed effect indeed is the effect of T, which would be in line with its pharmacodynamics profile and animal model data. On the other hand, the number of patients is actually modest and they were all recruited at three specialized centers in one city. The severity of GAD was limited to 32 score points (HAM-A extends to a maximum of 56) (6), hence they might not be really representative of the population of patients with GAD. Also, the proportion of responders (≥50% HAM-A reduction) (5) is considered the clinically more relevant outcome in the treatment of GAD. Overall, considering the entire context, elements of the trial (ie, the “entire *a priori* model”) and pre-existing knowledge about T, the observed effect supports the conclusion that T indeed has a potential to alleviate GAD in a short-term treatment. This does not mean that at this point it should be considered an “effective treatment” (ie, that indeed there is a true effect of T) – this is yet to be evaluated (short-term/long-term, wider target population), particularly, the size of its effect is yet to be estimated. In the scenario 2, all the above considerations about T and this hypothetical trial are the same. There is only one minor difference: HAM-A reduction with T is slightly less (by around 0.5 score points) than in the scenario 1, and the reduction with PBO is by 0.1 score point higher (Table 1). The effect is “in favor of T,” with a practically identical width of the 95% CI around it as in the scenario 1, but the point-estimate is by some 0.5 score points lower and the lower limit of the 95% CI falls below 0 (Table 1). The *t*-score is 1.85 (Table 1), corresponding to the 96.7th percentile of the respective *t*-distribution (Figure 1C) and resulting in *P* = 0.066 (Table 1). What should the conclusion based on this scenario be? A large part of the ASA documents (1,2) and the accompanying discussion aimed to provide argument that would discourage the unjustifiably prevailing practice of “dichotomization” of the *P* value and all the consequences. The whole concept of frequentist statistical tests was not conceived in this fashion (1-4). It is beyond our reach and scope to go any further into this topic – the “source” material is freely available to any interested reader. In respect to the hypothetical scenario 2, we feel free to state that the overall conclusion about T should be the same as in the scenario 1 but with somewhat greater uncertainty about it.

So, what about *P*?

With the understanding that is available to us as lay persons, it appears that there is “nothing wrong” with the essence of hypothesis tests and *P* values, rather it is important how we “treat it,” interpret it, and contextualize it.

**Table 1:** Two hypothetical outcomes of the trial of a new treatment vs placebo (see Box for outline). Reduction in Hamilton Anxiety rating scale (HAM-A) is shown with a positive sign, for clarity. Higher values = greater reduction*†

	Scenario 1		Scenario 2	
	treatment	placebo	treatment	placebo
N	88	92	88	92
Mean HAM-A reduction	13.1 (SD 9.9)	9.8 (SD 9.5)	12.6 (SD 10.0)	10.0 (SD 9.8)
Baseline-adjusted	13.01 (SE 1.03)	9.89 (SE 1.00)	12.64 (SE 1.03)	9.97 (SE 1.01)
Mean difference T-placebo	3.12 (SE 1.44), 95% CI 0.29, 5.96		2.67 (SE 1.44), 95% CI -0.17, 5.52	
The null hypothesis test	*t* = 2.18, d.f. 177, *P* = 0.0308		*t* = 1.85, d.f. 177, *P* = 0.066	

*SD – standard deviation; SE – standard error; CI – confidence interval.

†Individual data on HAM-A reduction and baseline scores were sampled from a normal distribution. The latter was restricted by the inclusion criteria of HAM-A score between 20 and 32. For analysis, we used SAS 9.4 for Windows (SAS Inc., Cary, NC), proc mixed with treatment and baseline HAM-A as effects, with maximum likelihood estimation.
